# Typical and Atypical Presentations of Appendicitis and Their Implications for Diagnosis and Treatment: A Literature Review

**DOI:** 10.7759/cureus.37024

**Published:** 2023-04-02

**Authors:** Sophia Echevarria†, Fatima Rauf†, Nabeel Hussain†, Hira Zaka, Umm-e- Farwa, Nayab Ahsan, Alison Broomfield, Anum Akbar, Uzzam Ahmed Khawaja

**Affiliations:** 1 Surgery, Universidad Mayor de San Simón, Cochabamba, BOL; 2 Internal Medicine, Rawalpindi Medical University, Rawalpindi, PAK; 3 Internal Medicine, Saba University School-Medicine, Devens, USA; 4 Neurosurgery, Jinnah Postgraduate Medical Centre, Karachi, PAK; 5 Surgery, Jinnah Sindh Medical University, Karachi, PAK; 6 Internal Medicine, Quaid-e-Azam Medical College, Bahawalpur, PAK; 7 Family Medicine, Spartan Health Sciences University, Vieux Fort, LCA; 8 Pediatrics, University of Nebraska Medical Center, Omaha, USA; 9 Pulmonary and Critical Care Medicine, Jinnah Medical and Dental College, Karachi, PAK; 10 Clinical and Translational Research, Dr Ferrer BioPharma, South Miami, USA

**Keywords:** appendix, therapeutic interventions, diagnostic modalities, typical appendicitis, atypical appendicitis

## Abstract

Appendicitis, an acute inflammation of the appendix, affects all demographic groups and exhibits various incidences and clinical manifestations. While acute appendicitis typically presents with colicky periumbilical abdominal pain that localizes to the right lower quadrant, atypical presentations are more common in children, geriatric, and pregnant patient populations, leading to delays in diagnosis. Clinical evaluation, clinical scoring systems, and inflammatory markers are commonly used, but their limitations have led to the increased use of diagnostic imaging in patients suspected of appendicitis. Acute appendicitis is managed by non-operative and operative management, depending on whether it is uncomplicated or complicated. Developing diagnostic pathways to improve outcomes and reduce complications is crucial. Although medical advancements have been made, diagnosing and managing appendicitis can be challenging, mainly when patients are present atypically. This literature review aims to comprehensively review typical and atypical presentations of appendicitis and their current implications for diagnosis and treatment modalities in pediatric, adult, pregnant, and geriatric patient populations.

## Introduction and background

The appendix has a diverse evolutionary history, and its purpose has been debated and researched for many years [1]. Thus, according to new research, the appendix contributes to the body's defense by promoting mucosal immunity against pathogens and balancing intestinal microbes [1]. The inflammation of the vermiform appendix is known as appendicitis [2]. The incidence of appendicitis is estimated to be around 100 cases per 100,000 individuals per annum, with a consistent occurrence rate in Western nations and an upward trend in developing regions. This condition commonly affects the age range between 5 and 45 years [3]. The incidence of morbidity and mortality associated with appendicitis varies based on demographic factors, with a higher prevalence observed in the pediatric population and a peak in adolescents.

In contrast, mortality rates are higher in the elderly [4]. Some authors have also reported a gender predisposition in all ages, slightly higher among males, with a lifetime incidence of 8.6% for men and 6.7% for women [2]. However, women tend to have a higher appendectomy rate because of various gynecological conditions that emulate appendicitis [5,6].

According to population-based ethnicity statistics, appendicitis is more common in white-non-Hispanic and Hispanic groups and less common in Blacks and other race-ethnicity groups [7,8]. However, data show that minority groups are at a higher risk of perforation and complications [9,10]. The reasons are still unclear due to the limitations of studies but have been predicted based on low socioeconomic background, inadequate health access relating to insurance status, and unequally equipped medical centers leading to delayed management [11]. Although the diagnosis of appendicitis is usually straightforward, it can be challenging in children, pregnant and the elderly, which can present with late or atypical features, leading to more severe or lethal complications.

Although the etiology of appendicitis is not always determined, it is thought to be multifactorial, including but not limited to environmental influences and genetic factors [7]. Predominantly the pathogenesis starts with luminal obstruction as a blind-ended tube connected to the cecum; the most common cause is a fecalith. Another leading factor, especially in children, is lymphatic hyperplasia, which can be caused by genes due to the overgrowth of lymphoid tissue in the submucosa or stimulation from influenced viral infections, increasing lymphatic size and occlusion of the lumen [7,12]. Other causes include calculi, seeds, parasites such as Enterobius vermicularis (pinworm), as well as some rare tumors, whether benign (mucinous tumors) or malignant (adenocarcinoma, neuroendocrine tumors) [13,14].

**Figure 1:**
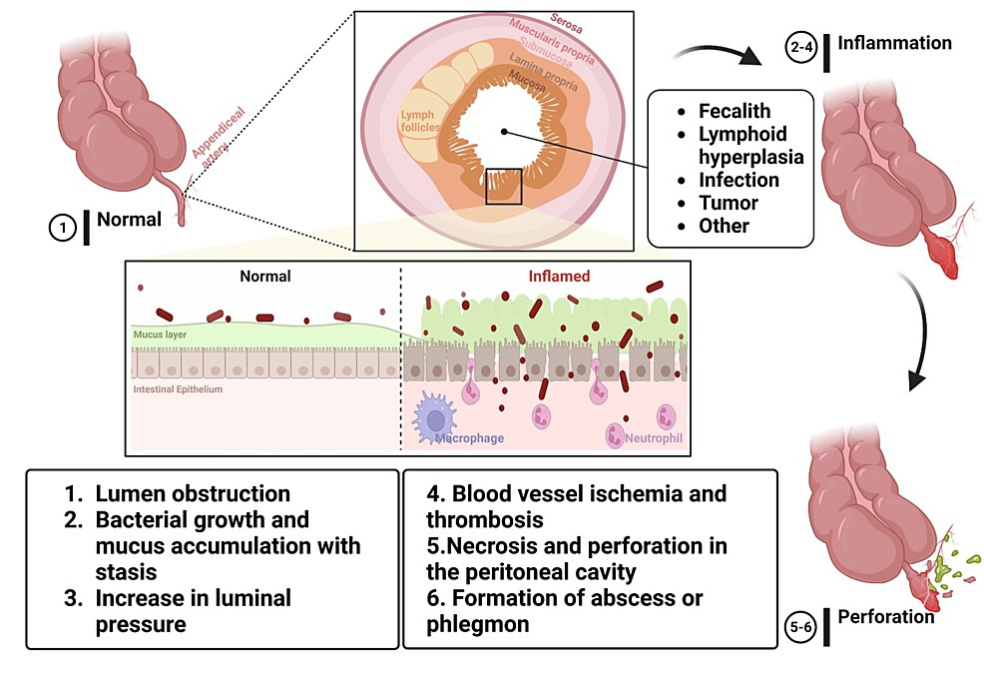
Pathogenesis of Appendicitis. (Created with bioRender.com)

Considering that the intestine constantly secretes mucus, when the flow is disrupted, the intraluminal and intramural pressure increases, causing appendiceal distension and pain due to the sensitivity of the visceral nerve fibers. Entering a vicious cycle, mucus and intestinal bacteria accumulate, activating white blood cells to invade the lumen (Figure 1).

The severity of appendicitis inflammation determines the presentation of signs, symptoms, and complications, with progressive edema and ischemia potentially leading to thrombosis of surrounding blood vessels, weakening of the epithelial wall, and necrosis, ultimately resulting in perforation and potentially fatal peritonitis, although the omentum may sometimes form a peri-appendiceal abscess or phlegmon; alternatively, appendicitis may resolve spontaneously or with antibiotics, resulting in either recurrent episodes or spontaneous alleviation [7,13,15].

Limited research exists on the risk factors associated with acute appendicitis. Nevertheless, certain factors that could potentially influence the likelihood of developing the condition include demographic factors such as age, sex, family history, and environmental and dietary factors [6]. Studies indicate that acute appendicitis can affect individuals of all ages, although it appears to be more prevalent among adolescents and young adults, with a higher incidence observed in males [16,17]. Like many other diseases, family history plays a significant role in acute appendicitis; based on the available data, individuals with a positive familial history of acute appendicitis are at an increased risk of developing the condition [18]. A few dietary risk factors are associated with appendicitis, such as a diet low in fiber, increased sugar intake, and decreased water consumption [19-22]. Environmental factors implicated in developing appendicitis include exposure to polluted air, allergens, cigarette smoke, and gastrointestinal infections [21,23-25].

New evidence suggests a potential correlation between elevated temperatures and acute appendicitis, proposing that high temperatures could augment the likelihood of developing the condition through dehydration [21]. Other associations of acute appendicitis include SARS-CoV2; it is reported that patients of acute appendicitis have more chance of having unrecognized COVID-19; therefore, it is recommended to test patients for SARS-CoV2 who present with acute appendicitis [26]. Studies have further shown that patients with psychiatric disorders who are administered high daily doses of antipsychotic medication are at an elevated risk of experiencing complicated appendicitis [27].

The typical presentation of appendicitis is characterized by epigastric pain that initially arises around the umbilicus and subsequently migrates towards the right lower quadrant of the abdomen, accompanied by nausea, vomiting, loss of appetite, and low-grade fever. However, atypical clinical features of appendicitis may also occur, leading to potential delays in diagnosis and management. Consequently, this medical literature review aims to synthesize available data on common and uncommon clinical presentations of appendicitis and evaluate their impact on diagnostic and therapeutic approaches.

## Review

Signs and symptoms

Clinical presentation of acute appendicitis depends on various factors, including the patient's age, duration of onset of symptoms, and anatomical variation of appendiceal position [28]. It can be classified into typical and atypical symptoms.

Typical Signs and Symptoms of Appendicitis

In children, appendicitis has a variability in presenting complaints according to age groups [29]. It is scarce and difficult to diagnose in neonates and infants [30]. They usually present with abdominal distension, vomiting, diarrhea, palpable abdominal mass, and irritability [31]. On physical examination, they are often dehydrated, hypothermic, and in respiratory distress, which makes a diagnosis of appendicitis unlikely for the clinician. Pre-school children up to 3 years of age usually present with vomiting, abdominal pain, mainly diffuse fever, diarrhea, difficulty walking, and stiffness in the right groin [32]. Assessment may reveal abdominal distension, rigidity, or a mass per rectal examination [12]. Children aged 5 years and above are more likely to present with classic symptoms, including migratory abdominal pain, anorexia, nausea, and vomiting. Clinical evaluation reveals pyrexia and tachycardia, decreased bowel sounds, and tender right lower quadrant favor the likelihood of diagnosis in this age group [33]. The presentation of acute appendicitis in younger children is usually atypical, with overlapping symptoms mimicking other systemic disorders, often leading to missed diagnosis and complications leading to morbidity [12]. Moreover, younger age is a well-known risk factor for poor outcomes due to complicated appendicitis [34].

**Figure 2:**
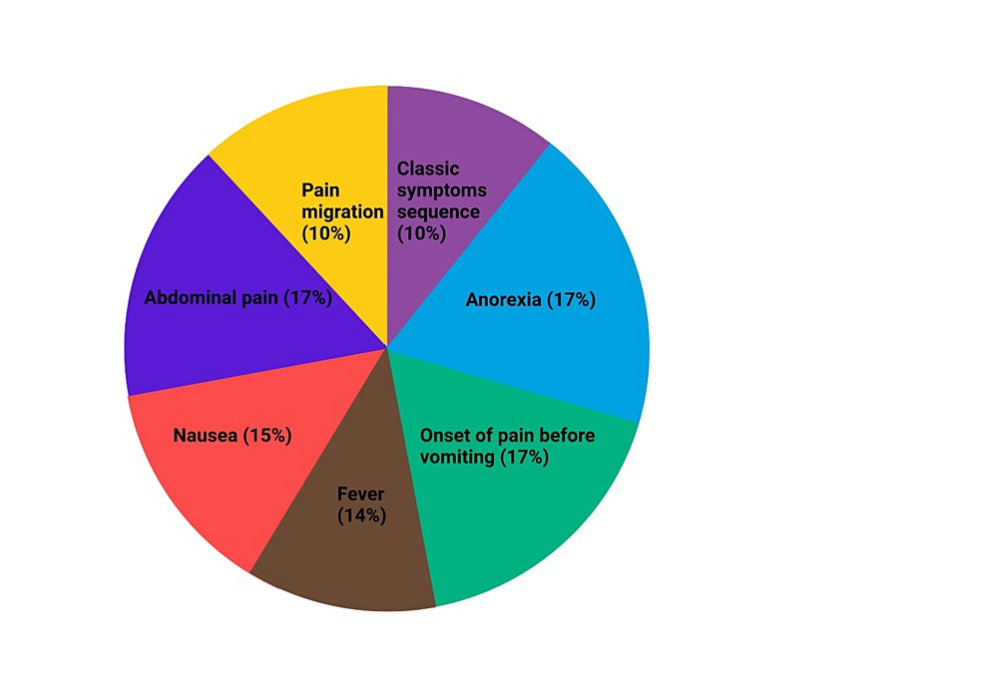
Common Symptoms of Appendicitis in Adults (Created with bioRender.com)

A typical presentation of appendicitis in adults includes migratory right iliac fossa pain, anorexia, nausea with or without emesis, fever, and localized muscle rigidity/ generalized guarding [35-39]. Classic symptom sequence is vague periumbilical pain to anorexia/nausea/unsustained vomiting to migrating pain to the right lower quadrant to low-grade fever. The most common presentation with frequency is shown in Figure 2 [36,40].

Previous studies on signs and symptoms show strong evidence of the association of migratory pain with appendicitis [1,28]. Initially, there is diffuse pain as the appendix receives visceral innervation, but as inflammation increases, the parietal peritoneum involves somatic innervation [1]. This leads to localizing the pain origin in RIF. Approximately 50- 60% of patients with appendicitis show the first sign of peri-umbilical pain localizing to the right lower quadrant within 24 hours. About 80 - 85 % of patients report anorexia, nausea, and fever [40]. The commonly used diagnostic signs for appendicitis are McBurney's, Rovsing's, Psoas, and Obturator. McBurney's sign involves tenderness in the lower quadrant of the abdomen, while Rovsing's sign is characterized by pain in the right lower quadrant upon palpating the left lower quadrant. The Psoas sign entails abdominal pain in the right lower quadrant with passive right hip extension.

In comparison, the Obturator sign involves pain in the right lower quadrant with passive right hip flexion followed by a right hip internal rotation. These signs have varying sensitivity and specificity in diagnosing appendicitis [28,36,40,41]. Guarding, rebound tenderness, and rigidity are additional clinical signs to assess appendicitis. These signs have sensitivity and specificity ranging from 39-74% and 57-84% in diagnosing appendicitis [40].

The clinical presentation of appendicitis can also vary based on the anatomical location of the inflamed appendix [1,40]. Anatomical-related symptoms may include diarrhea and rectal and vaginal irritation. The three major types of appendicitis based on anatomy are the Rectocecal/Retrocolic (75%), Sub-caecal Pelvic (10%), and Pre-ileal and Post-Ileal (5%) types. A positive psoas sign is commonly observed in these cases. In Sub-caecal pelvic appendicitis cases, supra-pubic pain and urinary frequency may predominate, and positive rectal or vaginal tenderness and Obturator signs are common. Pre and Post-Ileal appendicitis may present with vomiting as the primary symptom, and diarrhea may result from irritation of the distal ileum.

With age, as the anatomy of the appendix changes, elderly patients do not usually present with all typical signs and symptoms of acute appendicitis, are RIF pain, fever, anorexia vomiting [42]. In elderly patients with appendicitis, abdominal pain is the most common presenting symptom, with 75% of patients experiencing pain in the right lower quadrant. Other symptoms include vomiting in 27%, nausea, and anorexia in 9%, positive Rovsing signs in 10%, rectal temperature above 37.5°C in 71%, an abdominal mass on palpation in 7%, tenderness in the right lower quadrant or at McBurney's point in 9% and rebound pain with guarding in 40%. Additionally, 54% of elderly patients with appendicitis have both rebound pain and tenderness [41]. Acute appendicitis is the most common non-obstetric cause of surgery during pregnancy in emergency presentation. Classic symptoms include abdominal pain beginning at the periumbilical region and later shifting to the right lower quadrant, anorexia, nausea and vomiting, and elevated temperature [43].

The severity of acute appendicitis can be evaluated based on rebound tenderness and rigidity in the right lower quadrant, as these findings are highly correlated [35]. Rebound tenderness is an indicator of more severe histological inflammation of the appendix. The degree of histopathological severity has been found to significantly correlate with several clinical symptoms, such as pain migration to the right lower quadrant, loss of appetite, peri-umbilical pain, fever, and elevated white blood cell and polymorphonuclear leukocyte counts, as well as an increased proportion of polymorphonuclear leukocytes on differential analysis [35,44]. While rebound tenderness, coughing, and current signs are all sensitive indicators of parietal inflammation, there is no conclusive evidence of which signs are superior.

Sudden settling or decrease in the severity of pain is a possible indication of a ruptured appendix as the pressure within the appendiceal wall decreases. Physical examination may reveal a triad of symptoms, including tachycardia, low-grade fever (37.8°C), decreased bowel sounds, and increasing tenderness over time. A rigid abdomen is a clear perforation indicator and should be monitored to prevent further complications [26,33].

Atypical Signs and Symptoms of Appendicitis

In addition to the typical presentation of appendicitis, atypical signs, and symptoms may also be observed. These may include left-sided abdominal pain, which localizes to the left upper quadrant. While a left-side appendix is relatively rare, occurring in approximately 0.02% of the adult population, it is more likely to occur in individuals with gut malrotation or situs inversus [45]. Appendicitis is also associated with diarrhea as an atypical symptom in advanced appendicitis, especially in patients with inter enteric abscesses [46].

Children mainly present with vague symptoms making diagnosis difficult on history and examination. Atypical presentation of appendicitis in children may involve pain and tenderness along the entire right flank, extending from the right upper quadrant to the right iliac fossa. This could result from arrested caecal descent of the appendix, where the caecum is in the subhepatic position [47]. Adult males may present with atypical appendicitis symptoms, such as severe right hemiscrotal pain that later becomes mild diffuse abdominal pain. In contrast, females may present with genitourinary complaints such as tenderness in the femoral region with a mass and diarrhea [48,49]. In the elderly, appendicitis can present atypically as a strangulated inguinal hernia with non-specific symptoms [50].

Pregnant patients are more likely to present atypical complaints such as gastroesophageal reflux, malaise, pelvic pain, epigastric discomfort, indigestion, flatulence, dysuria, and altered bowel habits [43]. Furthermore, findings on physical examination are complex to illicit as the abdomen is distended, which increases the distance between the inflamed appendix and peritoneum, leading to masking of guarding/rigidity and decreased intensity of tenderness [43]. During advanced pregnancy, the appendix may be displaced cranially towards the upper abdomen due to the gravid uterus resulting in pain in RUQ [51]. However, regardless of gestational age, RLQ pain remains the most common clinical presentation of acute appendicitis in pregnancy [52]. Leukocytosis may not be a reliable indicator of acute appendicitis in pregnant women due to physiologic leukocytosis during pregnancy. Research studies have shown that pregnant women have a lower incidence of appendicitis than non-pregnant women. However, there is a higher risk of developing acute appendicitis during the second trimester [53].

Diagnosis

Diagnosing acute appendicitis (AA) is challenging as it presents varied symptoms and signs, and AA has been called a 'chameleon' of surgery for this reason [54]. Many diagnostic scoring tools have been introduced to diagnose AA efficiently. Recent guidelines recommend stratifying patients into low, intermediate, and high-risk groups based on clinical parameters and laboratory investigations to guide further management [55,56]. In addition to verifying the diagnosis, assessing appendicitis severity classification is crucial for clinicians to determine the appropriate management strategies, whether operative or non-operative.

Clinical parameters in the Diagnosis of Acute Appendicitis

The most common symptoms of acute appendicitis are umbilical pain migrating towards the right iliac fossa, anorexia, and nausea with or without vomiting [57]. Common signs include increased temperature, Mc Burney's sign (tenderness at Mc Burney's point), rebound tenderness, Rovsing sign (tenderness in a right iliac fossa on palpating left iliac fossa), psoas sign (pain in a right iliac fossa on passive extension of the right hip joint) and obturator sign (pain in a right iliac fossa on internal rotation of right hip joint with flexed hip and knee joint) [28]. However, the classic presentation of acute appendicitis is present in only 6% of patients, and atypical presentation is common [58].

Biochemical Parameters in the Diagnosis of Acute Appendicitis

Biochemical markers such as total leucocyte count (TLC) and C- reactive protein (CRP) have been widely used in diagnosing acute appendicitis and differentiating complicated and uncomplicated appendicitis. A normal TLC and CRP combination has a high sensitivity and negative predictive value [59]. Newer markers such as absolute neutrophil count, calprotectin (CP), serum amyloid A (SAA), and myeloid-related protein 8/14 (MRP 8/14) have also exhibited high sensitivity and negative predictive value [60-62]. Hyperbilirubinemia and hyponatremia have also been studied to predict complicated appendicitis [63,64]. Delta neutrophil index has been studied as a reliable biomarker in the elderly [65].

Role of Scoring Systems in the Diagnosis of Acute Appendicitis

Clinical and laboratory parameters alone have little diagnostic accuracy for acute appendicitis. Different scoring systems have thus been proposed for correct diagnosis and risk stratification of patients. Alvarado scoring system was initially used in diagnosing AA, and it has high sensitivity but low specificity [66]. Other scoring systems include Appendicitis Inflammatory Response Score (AIRS), the Adult Appendicitis Score (AAS), and RIPASA (Raja Isteri Pengiran Anak Saleha Appendicitis) score. Pediatric Appendicitis Score (PAS) and Pediatric Appendicitis Laboratory Score (PALabS) have been studied in children. AIRS and AAS have been recommended to diagnose acute appendicitis in adults. However, diagnosis solely based on scoring systems is not recommended in children [55]. Patients are stratified into three groups based on scores. The low-risk group is subjected to further investigations, and the intermediate-risk group is subjected to cross-sectional imaging. The high-risk group is taken to surgery without cross-sectional imaging [55,56].

Role of Imaging in the Diagnosis of Acute Appendicitis

Imaging modalities for diagnosis of acute appendicitis include ultrasonography (USG) and cross-sectional imaging such as contrast-enhanced CT scan (CECT) and magnetic resonance imaging (MRI). CECT scan is considered the gold standard in diagnosing AA. However, USG is recommended as the first-line imaging modality for diagnosing children and adults. In patients with equivocal USG findings, a low-dose CECT scan is recommended in patients with suspected appendicitis [67]. In high-risk groups based on clinical scoring, upfront surgery is recommended without cross-sectional imaging in patients under 40 [55,56]. This recommendation has sparked intense debate, with some clinicians proposing routine CT scans before surgery. A routine CT scan is associated with a far lower rate of negative appendectomies than selective imaging [68]. CT scans and MRI have higher sensitivity and specificity in diagnosing AA [69,70]. However, these are not recommended as first-line imaging modalities due to high radiation exposure and cost. Conditional use of cross-sectional imaging modalities in the presence of equivocal USG findings seems more practical [55,56]. MRI is useful in pregnant females and has high sensitivity and specificity; however, USG is still recommended as the first-line imaging modality. Moreover, negative MRI does not exclude AA, and surgery is recommended in the presence of high clinical suspicion [55,71].

Role of Diagnostic Laparoscopy in the Diagnosis of Acute Appendicitis

Diagnostic laparoscopy is recommended in patients with an atypical presentation, equivocal imaging findings, and persistent or worsening symptoms. It can serve as a diagnostic and therapeutic tool [55]. It can effectively diagnose and treat AA and reduce the risk of complications [72].

Differentiation Between Uncomplicated and Complicated Acute Appendicitis

Since the treatment of uncomplicated and complicated appendicitis differs, it is essential to differentiate between them. However, no diagnostic modality sufficiently differentiates between them [7]. Clinical scoring systems can predict complicated appendicitis to a certain degree. Similarly, biochemical markers can distinguish between uncomplicated and complicated appendicitis [63,73,74]. CT scan findings of complicated appendicitis include Extraluminal appendicolith, abscess, appendiceal wall enhancement defect, extraluminal air, ileus, peri appendiceal fluid collection, ascites, intraluminal air, and intraluminal appendicolith; however, their sensitivity is limited. The presence of appendicolith is associated with the failure of non-operative management [75]. Similarly, a triad of CRP >60 g/L, WCC >12 × 10^9^/L, and age >60 years are associated with complicated appendicitis [76].

Management of appendicitis

Management of appendicitis is broadly classified into non-operative and operative.

Non-operative Management of Appendicitis

The goal of non-operative management (NOM) is for patients to avoid surgery through antibiotics [77]. Early studies in the 1950s reported successful treatment of acute appendicitis with antibiotics alone, recommending the treatment of appendicitis with symptoms lasting less than 24 hours [78,79]. In recent years, there has been renewed interest in NOM of uncomplicated acute appendicitis, with several studies reporting successful treatment of approximately 65% of cases using antibiotics alone. However, studies such as the APPAC, ACTUAA, and meta-analysis trials have shown mixed results, with short and long-term failure rates of NOM ranging from 11.9% to 39.1% [80-84]. Additionally, studies on using NOM for complicated appendicitis are limited but have shown that while it can be successful, it is associated with increased readmission rates and longer hospital stays [85,86].

The 2016 Jerusalem Guidelines and EAES guidelines recommend appendectomy as the preferred treatment for acute appendicitis and caution against the routine use of NOM [87,88]. However, the 2020 Jerusalem Guidelines suggest NOM as a first-line treatment for uncomplicated AA, with careful counseling regarding the possibility of failure in adults and children [55]. Recent evidence shows increased readmission rates, treatment failure, and complications with NOM [89,90]. While few studies have examined the quality of life (QOL) after either treatment, the CODA trial showed no significant difference between NOM and appendectomy [90,91]. Nonetheless, current recommendations remain unchanged.

There is currently no established antibiotic regimen for the non-operative management of acute appendicitis. Based on a recent meta-analysis, the optimal antibiotic regimen for non-operative management was recommended to be carbapenems [92]. For complicated acute appendicitis with phlegmon or abscess, non-operative management with antibiotics or percutaneous drainage as an adjunct to antibiotics is recommended as the first-line therapy [93-95]. Early laparoscopic appendectomy is recommended to decrease additional interventions and readmissions [96,97]. Interval appendectomy after successful non-operative management is not recommended for children and adults < 40 unless recurrent symptoms occur. Non-operative management is not recommended for elderly patients, obese patients, patients with comorbidities, and pregnant patients due to increased morbidity and mortality [55].

Operative Management of Appendicitis

The primary approach for treating appendicitis has been surgical, which was first suggested by Reginald Heber Fitz in 1886 and later refined by Mc Burney in 1889 through emergency laparotomy and a muscle-splitting incision now known as Mc Burney's incision [98-100]. Over the years, surgical management has evolved from open appendectomy to laparoscopic appendectomy and, more recently, to single-incision surgery, endoscopic retrograde appendicitis therapy, and natural orifice transluminal endoscopic surgery.

Laparoscopic vs. Open Appendectomy

Open appendectomy was the traditional surgical approach for acute appendicitis before the advent of laparoscopic appendectomy in 1981 [100]. Initially, laparoscopic appendectomy was reserved for uncomplicated cases due to concerns about an increased incidence of intra-abdominal abscess in complicated cases. However, recent evidence shows no significant difference in intra-abdominal abscess formation between open and laparoscopic approaches [101,102]. Laparoscopic appendectomy offers several advantages, including shorter hospital stays, lower rates of readmission and complications, and reduced occurrence of surgical site infections [103]. Guidelines now recommend laparoscopic appendectomy for all cases of acute appendicitis, including complicated cases, in all age groups [7,55].

Laparoscopic Single Incision Surgery

Previous studies conducted a meta-analysis of randomized controlled trials comparing single-incision laparoscopic surgery (SILA) with conventional multi-incision laparoscopic appendectomy (CLA). They demonstrated no significant difference between SILA and CLA regarding complication rates, length of hospital stay, or post-operative pain. Regarding operative time and analgesic requirements, CLA demonstrated significant superiority over SILA, whereas SILA yielded significantly better cosmetic outcomes [104,105]. Recent evidence recommends conventional multi-incision laparoscopic appendectomy (CLA) over single-incision laparoscopic surgery (SILA) [55].

Endoscopic Retrograde Appendicitis Therapy (ERAT)

Endoscopic retrograde appendicitis therapy (ERAT) is a minimally invasive procedure for treating appendicitis, with reported success rates ranging from 92.1% to 99.36% and low complication rates [106-108]. ERAT has also demonstrated advantages over laparoscopic appendectomy, including better pain outcomes and shorter hospitalization. ERAT is particularly effective in children, with significantly higher success rates than antibiotics-only treatment [109,110]. However, there currently needs to be a consensus regarding the preferred treatment approach for acute appendicitis, and further research is needed to compare ERAT with other treatment modalities.

Natural Orifice Transluminal Endoscopic Surgery (NOTES)

In 2008, Palanivelu performed the first natural orifice transluminal appendectomy (NOTES) using a transvaginal approach [111]. Since then, successful cases of NOTES appendectomy have been reported using different routes, such as transgastric, transvesical, trancolonic, and transvaginal routes [112-115]. NOTES appendectomy can be performed solely or combined with laparoscopic visualization, called hybrid-NOTES. However, the routine application of NOTES appendectomy is controversial and lacks recommendations.

Management of Appendicitis With Atypical Presentation

Diagnosing and managing patients presenting with atypical symptoms of acute appendicitis can be challenging, particularly in children, pregnant females, and elderly patients [116,117]. Diagnostic laparoscopy effectively diagnoses and treats acute appendicitis identifies alternative diagnoses, and avoids unnecessary surgeries [118,119]. Laparoscopy has helped children identify alternative diagnoses and avoid unnecessary appendectomies [120]. For elderly patients, laparoscopic appendectomy is recommended due to the higher incidence of complicated appendicitis [121]. Non-operative management is not preferred, and follow-up colonic screening is recommended. Pregnant females can undergo laparoscopic appendectomy without significantly impacting fetal loss or pre-term delivery [122]. In equivocal cases, cross-sectional imaging such as ultrasound, CT scan, or MRI is recommended. Diagnostic laparoscopy is recommended if symptoms persist despite normal imaging [55].

Discussion

Acute appendicitis is a prevalent cause of acute abdominal pain in the emergency department worldwide [123,124]. The condition involves acute inflammation of the vermiform appendix located at the tip of the cecum in the right lower quadrant [2]. The etiology of appendicitis is multifactorial, often resulting from lumen obstruction caused by a fecalith, lymphoid hyperplasia, malignancy, or parasites [2,7,12]. Pathogenesis involves increased intraluminal pressure due to lumen obstruction, leading to compromised blood supply, thrombus formation, ischemia, necrosis, gangrene, and possible perforation if medical attention is delayed [12].

The clinical diagnosis of appendicitis is typically based on characteristic symptoms, such as colicky abdominal pain, nausea, vomiting, and low-grade fever [40]. However, these symptoms are only present in about half of all cases, and the diagnosis of appendicitis can be challenging in certain patient populations [125]. In young children, atypical presentations may include abdominal distension, palpable masses, cellulitis of the abdominal wall, irritability, diarrhea, and pain with walking [12,43,126]. Atypically appendicitis in adult patients may present with diminished pain sensation, abdominal distension, and signs of peritonitis such as rigidity and guarding [127]. Pregnant women may exhibit unusual symptoms, such as right hypochondriac pain, dyspepsia, pelvic pain, flatulence, and changes in bowel movements [128]. Unusual presentations of acute appendicitis in patients at the extremes of age can present diagnostic challenges and may result in missed diagnoses, complications, and mortality. While the incidence of acute appendicitis generally decreases with age, the fatality rate is higher in elderly patients than in children [4]. Atypical presentations are more common in pregnant and elderly individuals due to various physiological factors and possible masking of symptoms by underlying malignancies [129]. Therefore, laboratory findings alone should not be relied upon for diagnosis, and imaging should be used with clinical evaluation [4]. These findings highlight the need for further research in these patient groups to improve diagnostic accuracy and reduce the risk of complications. The variability in presentation and non-specific symptoms in the general population can pose challenges for clinicians in accurately diagnosing acute appendicitis.

The management of appendicitis depends on whether it is uncomplicated or complicated by perforation, abscess, or phlegmon. Non-operative management, consisting of patient observation, oral intake restriction, and intravenous and oral antibiotics, is recommended for cases of uncomplicated appendicitis [55,130,131]. Percutaneous or transrectal drainage may be used in an abscess or phlegmon formation [94]. However, recent studies suggest that operative management is superior, and interval appendectomy is recommended after non-operative management to reduce the risk of future readmission for appendicitis [90,132]. Urgent surgery is recommended for cases of complicated appendicitis with generalized peritonitis. Laparoscopic appendectomy is generally recommended over open appendectomy due to its shorter length of stay, lower readmission rate, and lower overall complication rate [89,97]. Although there has been growing interest in alternative procedures such as laparoscopic single-incision appendectomy, endoscopic retrograde appendicitis therapy (ERAT), and natural orifice transluminal endoscopic surgery (NOTES), there are no current recommendations to support these procedures over conventional laparoscopic appendectomy [55,110,133].

## Conclusions

In conclusion, efficient triage and diagnostic planning for patients with suspected appendicitis are crucial in improving healthcare outcomes due to the diverse range of atypical presentations, which can result in delayed or missed diagnoses. Patient age, anatomical location of the appendix, and pregnancy status are key factors that can influence atypical presentations. Accurate diagnosis and optimal clinical management can be achieved through clinical scoring systems, laboratory findings, and imaging modalities. When used in combination, these tools can reduce the incidence of morbidity and mortality. Despite ongoing debate regarding the optimal approach for achieving curative outcomes, laparoscopic surgery remains the preferred treatment for simple and complex appendicitis. There is insufficient evidence to support alternative operative management options. However, non-operative management, which involves intravenous antibiotics followed by conservative oral therapy, remains viable for uncomplicated appendicitis. It is important to note that diagnostic and management approaches may evolve with advances in medical technology and accessibility. This literature review provides an overview of managing appendicitis with an atypical and atypical presentation in various patient populations, encompassing pediatric, adult, geriatric, and pregnant patients. It also outlines the latest recommendations for treating acute appendicitis with typical or atypical signs and symptoms in these populations. However, it is restricted by the inclusion of studies published only in the English language. Additionally, information regarding patient preferences in guiding treatment decisions is sparse, and there need to be more needs to be more studies reporting the long-term impact of treatment on patient quality of life.
